# Crosstalk enables mutual activation of coupled quorum sensing pathways through “jump-start” and “push-start” mechanisms

**DOI:** 10.1038/s41598-023-46399-z

**Published:** 2023-11-06

**Authors:** Joseph George Sanders, Hoda Akl, Stephen J. Hagen, BingKan Xue

**Affiliations:** Department of Physics, University of Florida, Gainesville, FL 32611 USA

**Keywords:** Dynamical systems, Regulatory networks

## Abstract

Many quorum sensing microbes produce more than one chemical signal and detect them using interconnected pathways that crosstalk with each other. While there are many hypotheses for the advantages of sensing multiple signals, the prevalence and functional significance of crosstalk between pathways are much less understood. We explore the effect of intracellular signal crosstalk using a simple model that captures key features of typical quorum sensing pathways: multiple pathways in a hierarchical configuration, operating with positive feedback, with crosstalk at the receptor and promoter levels. We find that crosstalk enables activation or inhibition of one output by the non-cognate signal, broadens the dynamic range of the outputs, and allows one pathway to modulate the feedback circuit of the other. Our findings show how crosstalk between quorum sensing pathways can be viewed not as a detriment to the processing of information, but as a mechanism that enhances the functional range of the full regulatory system. When positive feedback systems are coupled through crosstalk, several new modes of activation or deactivation become possible.

## Introduction

Many bacteria regulate and synchronize population-wide behaviors by exchanging diffusible chemical signals with other individuals of the same or different species within the community^1^. By secreting these chemical signals, known as autoinducers, and detecting their local concentrations, the bacteria can induce phenotypes collectively, in response to environmental and population conditions. These quorum-sensing regulatory pathways are usually sensitive to a variety of environmental and cross-species cues in addition to their own autoinducers, so that they control multiple phenotypic outputs in a complex fashion^2^.

Quorum sensing bacteria typically synthesize and detect more than one chemically distinct autoinducer, often with positive feedback controlling the rate of autoinducer production. The different autoinducers are detected by cognate receptors that drive regulatory pathways coupled to varying degrees^3^. The ability to sense more than one autoinducer is hypothesized to provide a number of potential benefits to a microbial species. It may offer advantages in interspecies interactions, including greater resistance to manipulation by other species^4^ or the ability for both interspecies and intraspecies communication^5^. Multiple signals may also allow temporal control of distinct phenotypes if different autoinducers accumulate at different rates^6,7^, or they may help infer physical conditions such as spatial confinement^8^, or provide advantages in quorum cheating^9^. Sensing through multiple signals and receptors in general may allow more sophisticated control of output dynamics of a sensing pathway^10^. But the ligand-specificity of quorum sensing receptors varies considerably among species and strains^4^; autoinducers employed by one organism often elicit a response from non-cognate receptor pathways in related variants or other microbial species. The lack of signal specificity allows interspecies “crosstalk” in bacterial communities^11^, a phenomenon that has been widely explored in the context of social behaviors such as kin discrimination, eavesdropping and facultative cheating^3^.

Crosstalk can also occur within a single species or strain. A pathway that senses one autoinducer may also be activated or inhibited by other autoinducers produced by the same organism. As these effects may occur through several different mechanisms, several definitions of crosstalk have arisen. In a system of two signal/receptor pairs that drive different promoters, and where specificity is poor, crosstalk has been characterized in terms of whether the lack of specificity resides in the ligand/receptor interactions or at the promoter-binding level^12^. It is also common, however, for pathways that detect multiple signals to funnel down to a fewer number of downstream outputs^10^. An extreme case is *Vibrio harveyi*, which senses three distinct autoinducers, each with its own dedicated sensor kinase; information from the three kinases is funneled into control of the same phosphorelay system^13^. Such funneled architecture has been described as crosstalk^14^. Here, we find it useful to define crosstalk as any mixing between two signaling pathways A and B that have their own signal inputs and outputs, but wherein signal A also modulates to some extent the output of receptor B, and vice versa. Crosstalk is a degree of coupling between two functional sensing pathways in the same organism^15–17^, and the strength of crosstalk lies along on a continuum from very strong (funneled) to very weak (orthogonal pathways).

Because crosstalk mixes information received from separate signals, it would appear likely to degrade the performance of a quorum sensing pathway. It is however a highly evolvable property that can be reduced or even eliminated through (for example) receptor design^4,18,19^. Therefore, although there exist several hypotheses for why bacterial species use multiple autoinducer signals, there is still little understanding of why crosstalk is common in quorum sensing systems, and how it affects the output behaviors of these networks, beginning at the level of an individual organism.

**Figure 1:**
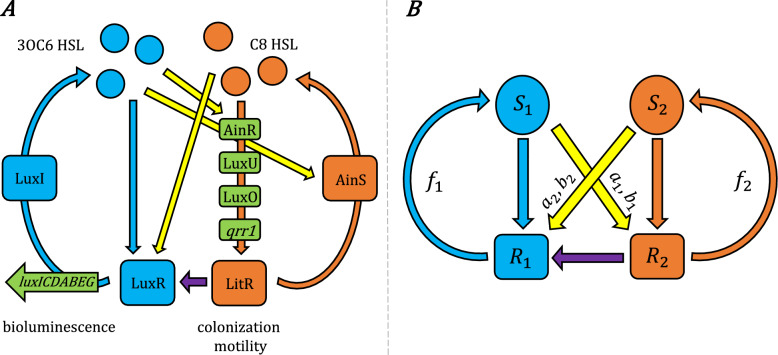
(**A**) The AinR/AinS and LuxR/LuxI quorum sensing pathways in *Vibrio fischeri*, which primarily control colonization traits and bioluminescence respectively, interact through several crosstalk mechanisms^20,22^. (A third pathway involving LuxS, LuxP/LuxQ and the autoinducer AI-2, coupled to the above through LuxU/LuxO, is not shown here.) The histidine kinase AinR detects its cognate autoinducer C8-HSL produced by AinS, initiating the LuxU/LuxO phosphorylation pathway. This pathway controls the expression of the regulatory RNA *qrr1*, a post-translational repressor of *litR*. In addition to controlling phenotypes related to motility and host colonization, LitR modulates production of LuxR, which is the intracellular receptor for the autoinducer 3OC6-HSL of the LuxI/LuxR pathway. LuxR becomes a transcriptional activator for the *lux* operon when bound either to its cognate signal 3OC6-HSL, produced by LuxI, or to the non-cognate C8-HSL. In addition, 3OC6-HSL interacts with LuxR to modulate activation of *ainRS*. Thus the AinR/AinS and LuxR/LuxI pathways both respond to each others’ autoinducers, while the AinR/AinS pathway also acts upstream of the LuxR/LuxI pathway through LitR. (**B**) The simplified model studied in this work captures the key elements of a quorum sensing system with crosstalk: Two signals ($S_1$, $S_2$) elicit their respective cognate responses ($R_1$, $R_2$), with crosstalk between them (yellow arrows) and an additional link between $R_1$ and $R_2$ that makes $R_2$ upstream of $R_1$. The signals are produced with positive feedback ($f_1$, $f_2$) from their respective responses. The crosstalk parameters $b_i$ and $a_i$ respectively define the strength of cross-binding (between each signal $S_i$ and its non-cognate receptor) and cross-activation (of the non-cognate response); see “Methods”.

Gram negative quorum sensing systems that employ autoinducers of the acyl homoserine lactone (HSL) type are particularly susceptible to crosstalk, as the HSLs are chemically similar and their cognate receptors typically respond to HSLs spanning a range of acyl chain lengths^11^. The quorum sensing system in the bacterium *Vibrio fischeri* is a model example^20^ with homologs in numerous other species^4^. We will focus on two pathways in that organism, LuxI/R and AinS/R, which are subject to several forms of crosstalk, shown in Fig. 1. The *lux* operon that controls bioluminescence is under immediate control of the LuxI/R pathway, a feedback loop in which LuxI is the synthase for the autoinducer 3-oxo-C6-homoserine lactone (3OC6-HSL) that interacts with the intracellular receptor LuxR to bind the *lux* promoter. However, the production of LuxR is modulated by LitR, which is controlled by a second, upstream quorum sensing pathway, AinS/R. AinS and AinR synthesize and detect respectively an autoinducer N-octanoyl-L-homoserine lactone (C8-HSL) to control LitR production. The LuxI/R and AinS/R pathways crosstalk through several mechanisms. The LuxR-3OC6-HSL complex is able to modulate expression of AinS, which encodes the C8-HSL synthase, by interacting with a *lux*-box-type binding site^20,21^. In addition, C8-HSL can also interact with LuxR to promote its binding to the *lux*-box. Thus, C8-HSL exerts an influence on the downstream LuxR/LuxI system via LuxR and LitR, while 3OC6-HSL influences the production of the C8-HSL synthase upstream.

In order to understand how the tuning of crosstalk strength affects a two-pathway quorum sensing system, we have analyzed a simplified model of the *V. fischeri* system. The model retains key features of two signals that primarily stimulate two responses, with crosstalk as well as positive feedback in autoinducer synthesis. We use this streamlined model to explore how different aspects of the crosstalk interact with feedback to reshape the steady state outputs that are available to the system.

## Model

We consider a model represented by the schematic in Fig. 1B, capturing the essential elements of crosstalk in the AinR/AinS and LuxR/LuxI quorum sensing pathways of *V. fischeri*. There are two signaling pathways, each of which produces a signal ($S_1$ or $S_2$) that induces a response ($R_1$ or $R_2$, respectively). The signal associated with each pathway is produced with positive feedback ($f_1$, $f_2$) from the response. One pathway ($R_2$, $S_2$) is functionally “upstream” of the other ($R_1$, $S_1$), in that response $R_1$ is dependent on $R_2$ (we do not call this link “crosstalk” because $R_1$ cannot function without it, so this link is not a tunable perturbation, see “Discussion” on “the meaning of crosstalk”). In addition, each of the two signals has some effect on the response of the other (“non-cognate”) pathway.

The elements of multiple signals, feedback and crosstalk are captured by the equations:

1a $$S_1= f_1 R_1$$

1b $$S_2= f_2 R_2$$

1c $$R_1= g_1 \, \frac{k_1 S_1^n + a_2 b_2 S_2^n}{1 + k_1 S_1^n + b_2 S_2^n} \, R_2$$

1d $$R_2= g_2 \, \frac{a_1 b_1 S_1^m + k_2 S_2^m}{1 + b_1 S_1^m + k_2 S_2^m}$$

These equations represent idealized steady states of a two-pathway sensing system (see detailed explanation in “Methods”). Although a real system in nature may never reach a steady state due to constant changes in environmental conditions over space and time, the steady states of a simplified model can help us understand generally how crosstalk shapes the system’s behavior. In these equations, $S_1$ and $S_2$ represent the concentrations of the two autoinducers; $R_1$ and $R_2$ represent the expression levels of the quorum-regulated genes of each pathway, including genes that encode the autoinducer synthases. Equations (1a, 1b) relate the signal concentrations to the regulated genes, due to positive feedback. Equations (1c, 1d) give the steady state response to the two signal levels. Among the parameters, $f_i$ is the feedback strength for each autoinducer, which depends on the rate of signal synthesis and the population density of cells; $g_i$ is the maximum expression level of $R_i$; $k_i$ is the interaction strength of a receptor with its cognate signal. For noncognate (crosstalk) interactions, the strength of binding and activation are described separately: $b_i$ (“binding”) captures the ability of a signal to competitively bind its non-cognate receptor; $a_i$ (“activation”) describes the efficiency of a signal, when bound to the non-cognate receptor, in cross-activating the non-cognate response. Finally, the exponents *n* and *m* represent the cooperativity of the signal response in each pathway.

We can simplify the equations by rescaling $S_1$ and $S_2$ to set $k_1 = k_2 = 1$, and rescaling $R_1$ and $R_2$ to set $g_1 = g_2 = 1$ (see “Methods”). Then the maximum value for the rescaled $R_1$ and $R_2$ is 1 (for $a_i \le 1$). Of the remaining parameters, $a_i$ and $b_i$ control the crosstalk strength. If the binding $b_i = 0$, then the signal from pathway *i* cannot elicit any response from the non-cognate pathway, so there is no crosstalk. On the other hand, if the activation $a_i = 0$, the signal from pathway *i* may bind to but not activate the response of the non-cognate pathway. For $a_i > 0$ there is potential cross-activation between the pathways; we assume $a_i \le 1$, which means the cross-activation by the non-cognate signal cannot be more efficient than the cognate signal. In most of what follows, we focus on the parameters $a_2$ and $b_1$, and set the other two parameters $a_1 = b_2 = 1$. That is, we focus on the case where $S_2$ interacts strongly with the noncognate receptor, but the complex is not necessarily an efficient activator for $R_1$. This is partly motivated by the example of *V. fischeri*, in which the effect of C8-HSL (analogous to $S_2$) on *lux* expression (analogous to $R_1$) is well known^23^, and 3OC6-HSL (analogous to $S_1$) has been shown to stimulate the *ainRS* pathway (analogous to $R_2$)^21^. We will further assume that $m = n$, and consider a range of values for the cooperativity *n*.

The responses $R_1$ and $R_2$ are not simply functions of $S_1$ and $S_2$ as in Eqs. (1c,1d), except in the special circumstance where signal levels are externally controlled. In natural settings the signals are tied to the responses through the feedback $f_1$ and $f_2$. As a result, the equilibrium values of all signals and responses are determined by the feedback strengths by solving Eqs. (1a–1d). Eliminating $S_1$ and $S_2$ from Eqs. (1c,1d) using Eqs. (1a,1b) and simplifying the parameters as described above, we arrive at two self-consistent equations for $R_1$ and $R_2$:

2a $$R_1= \frac{(f_1 R_1)^n + a_2 (f_2 R_2)^n}{1 + (f_1 R_1)^n + (f_2 R_2)^n} \, R_2$$

2b $$R_2= \frac{b_1 (f_1 R_1)^n + (f_2 R_2)^n}{1 + b_1 (f_1 R_1)^n + (f_2 R_2)^n}$$

We find the solutions to these equations using numerical solvers from the SciPy package for Python. We first solve a set of ODEs for which Eq. (2) are the equilibrium (see “Methods”). We integrate these ODEs for a sufficient amount of time that the variables come close to an equilibrium. Then, we use these values as initial guesses for a root solver to find the precise solutions. This method allows us to find multiple solutions if they exist and are stable, by using many random initial values in solving the ODEs. Once the solutions are refined using the root solver, we remove redundant solutions that have already been found (see “Methods” for details).

## Results

The solutions to our main Eqs. (2a, 2b) represent the responses $R_1$ and $R_2$ as functions of the feedback strengths $f_1$ and $f_2$. Increased feedback strength may correspond to the condition of high cell density, where the autoinducer is captured by neighboring cells rather than being lost to the environment. When the cell density reaches a certain level, a phenotypic response is triggered. Our goal is to see how this response is modulated by the crosstalk parameters $a_2$ and $b_1$. Without crosstalk, the pathways operate independently: response $R_i$ will be activated if the feedback $f_i$ reaches a certain level. Crosstalk allows the feedback $f_1$ not only to elicit the cognate response $R_1$ but also to influence the other response $R_2$, and vice versa. We will characterize such effects below.

### Crosstalk can both activate and inhibit non-cognate responses

**Figure 2:**
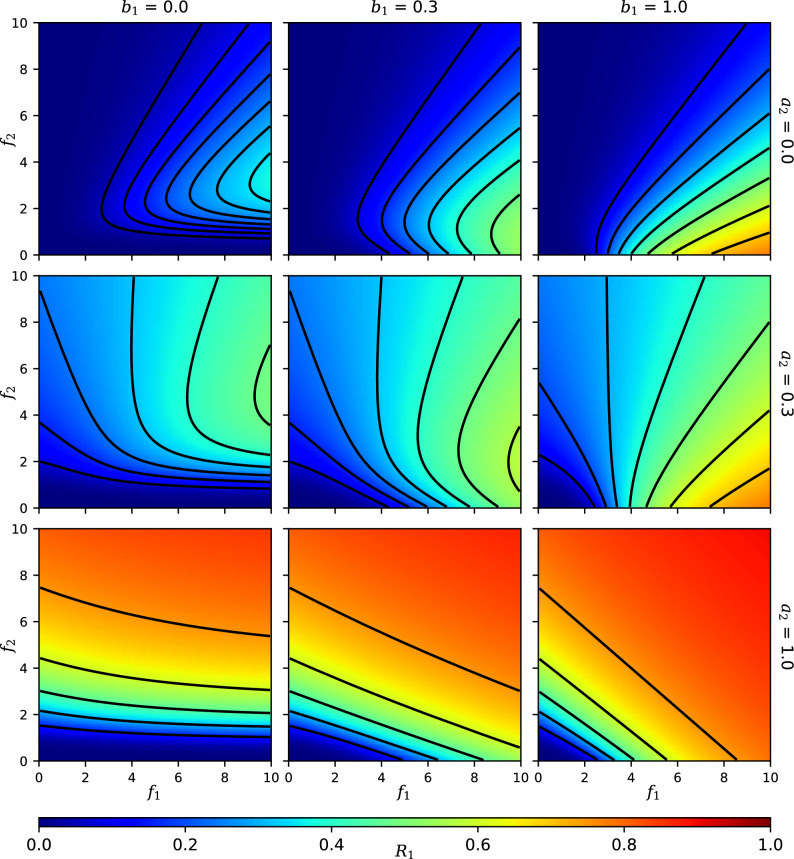
Heat maps showing the value of $R_1$ as a function of the feedback strengths $f_1$ and $f_2$. Rows correspond to different values of the downstream-directed crosstalk activation strength $a_2$, whereas columns correspond to values of the upstream-directed crosstalk binding strength $b_1$ (all panels have $a_1 = b_2 = 1$, $n = 1$). Black lines are contours of constant $R_1$.

Figure 2 shows a heat map of $R_1$ as a function of $f_1$ and $f_2$. $R_1$ is of interest as it is the most downstream element in our circuit (Fig. 1B), affected by both signals and the upstream response $R_2$. When both crosstalk parameters are at full strength, $a_2 = b_1 = 1$ (given that we also assume $a_1 = b_2 = 1$), $R_1$ is determined simply as a linear combination of $f_1$ and $f_2$ (Fig. 2 bottom-right panel). It means that $R_1$ is equally well activated by either one of the quorum signals, either directly by the cognate $S_1$ or through crosstalk by the non-cognate $S_2$. This strong crosstalk is analogous to what occurs in *V. harveyi* quorum sensing network, where three autoinducer inputs add linearly to give a single output^24^.

Figure 2 also shows how the strength of the crosstalk $a_2$ determines whether the non-cognate signal $S_2$ activates or inhibits the response $R_1$. Strong activating crosstalk $a_2$ (Fig. 2 bottom row) allows $R_1$ to increase with $f_2$. Weakly activating crosstalk, where $a_2$ is small (Fig. 2 top row), allows a large $f_2$ to inhibit $R_1$. This is because at the low $a_2$ limit cross-activation is inefficient, so that non-cognate binding ($b_2 = 1$) allows competitive inhibition of $R_1$ by $S_2$.

Another feature to note is that, when the crosstalk binding strength $b_1$ is very small (Fig. 2 left column) and $f_2$ is also small, no amount of $f_1$ can activate $R_1$. This is because $R_1$ relies on the upstream response $R_2$, which remains off under conditions of small $f_2$ and $b_1$. However, as $b_1$ increases (right column), part of the small-$f_2$ region can now be activated by $f_1$ alone: Crosstalk from the downstream signal $S_1$ to the upstream response $R_2$ can activate the downstream response $R_1$. In “Discussion” on “new motifs” we elaborate on this mechanism where crosstalk from the downstream signal activates both responses.

### Crosstalk can modulate the dynamic range of joint responses

**Figure 3:**
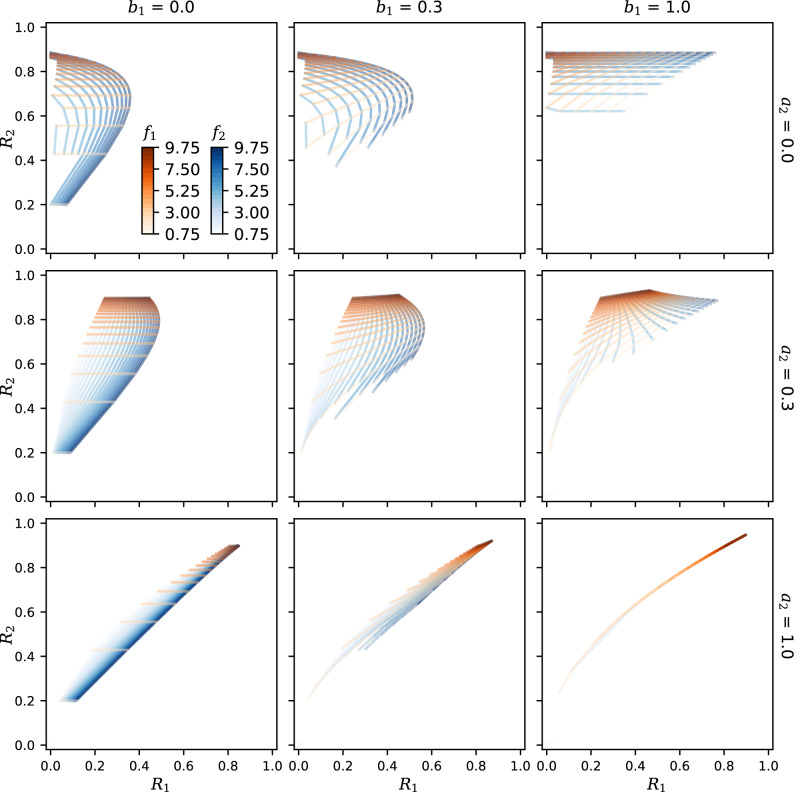
Mesh plots showing the steady state $R_1$ and $R_2$ at different feedback strengths. Orange curves represent constant $f_1$ values, and blue curves represent constant $f_2$ values. Rows and columns correspond to different values of the crosstalk parameters $a_2$ and $b_1$, respectively (with $a_1 = b_2 = 1$, $n = 1$).

To study the dynamic range of both responses $R_1$ and $R_2$ together, we make a parametric plot of their values as the feedback $f_1$ and $f_2$ are varied (Fig. 3). This creates a mesh of possible solutions that deforms as the crosstalk strengths $a_2$ and $b_1$ change. The mesh lies in the upper left half of each panel, because $R_1 \le R_2$ as a result of Eq. (2a): the downstream response $R_1$ relies on the upstream $R_2$. $R_1$ and $R_2$ show greatest range and span a broader, two-dimensional region of the graph when crosstalk is weak, i.e., with $a_2$ small (Fig. 3 top row). As the crosstalk strength increases, the $R_1,R_2$ responses become more tightly coupled and span a reduced area. The effect is most apparent when both $a_2$ and $b_1$ approach 1 (Fig. 3 bottom right), for which the 2D mesh collapses toward a single curve. $R_1$ and $R_2$ are then tied together, and are both linear in $f_1$ and $f_2$ as seen from Fig. 2 (bottom right; see also Fig. S1).

### Crosstalk can facilitate new mechanisms of activating responses

**Figure 4:**
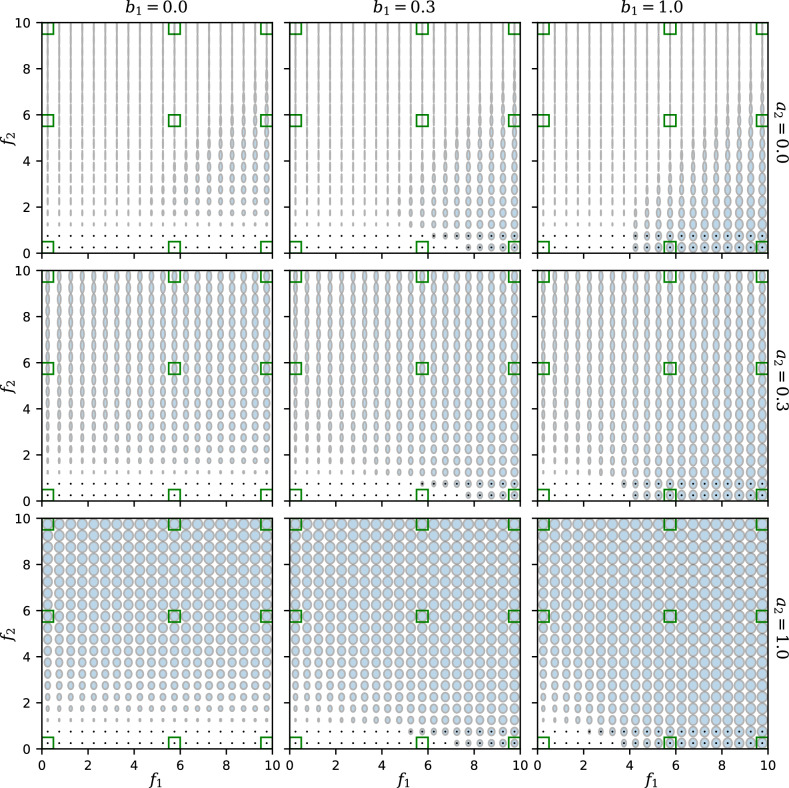
“Ellipse plots” showing the responses $R_1$ and $R_2$ simultaneously as functions of the feedback strengths $f_1$ and $f_2$. The width and height of each ellipse represent the values of $R_1$ and $R_2$, respectively, at a given point in the $(f_1, f_2)$ plane. A black point superimposed on an ellipse indicates that the trivial state $R_1 = R_2 = 0$ is also stable. Rows and columns represent different values of the crosstalk parameters $a_2$ and $b_1$, respectively (with $a_1 = b_2 = 1$, $n = 1$). The $f_1$ and $f_2$ values marked in green are further explored in Fig. 5.

**Figure 5:**
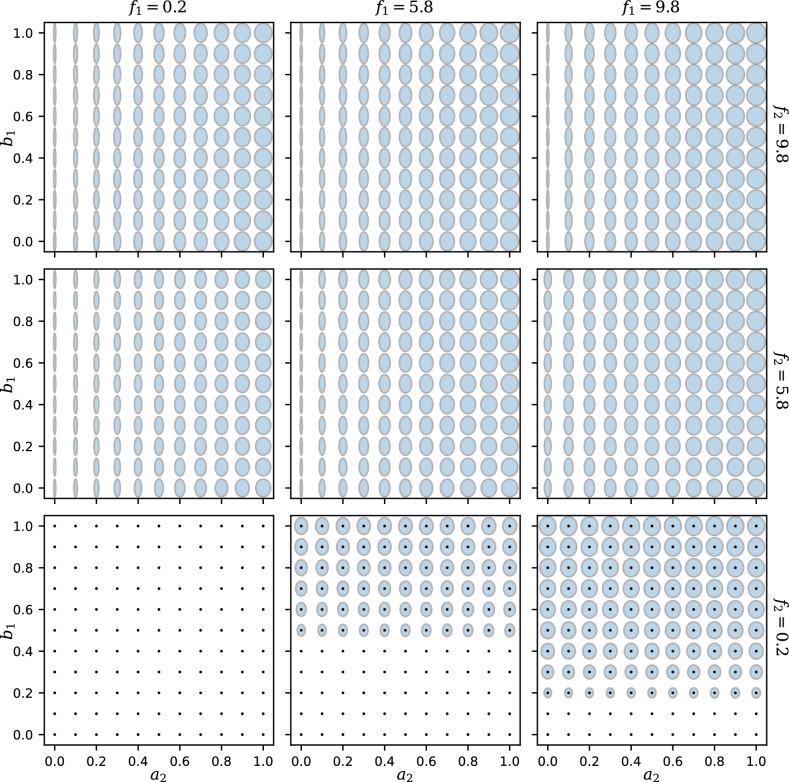
Dependence of the responses $R_1$ and $R_2$ on the crosstalk parameters $a_2$ and $b_1$. With the same parameters as in Fig. 4, the width and height of each ellipse represent the values of $R_1$ and $R_2$, respectively. Rows and columns here represent different combinations of the feedback $f_1$ and $f_2$ (marked green in Fig. 4). A black point superimposed on an ellipse indicates that the trivial state $R_1 = R_2 = 0$ is also stable.

To visualize how both responses $R_1$ and $R_2$ depend on the feedback $f_1$ and $f_2$, we create “ellipse plots” in Fig. 4, in which an array of ellipses displays the values of both $R_1$ and $R_2$ at different positions in the $(f_1, f_2)$ plane. For each ellipse, the horizontal axis is proportional to the value of $R_1$, and the vertical axis is proportional to $R_2$. Thus, the width of each ellipse in each panel of Fig. 4, as a function of $f_1$ and $f_2$, matches the $R_1$ value shown in the heat map of Fig. 2, while the height of each ellipse represents $R_2$ (Fig. S1). Where there are multiple stable solutions at the same $(f_1, f_2)$ point, we overlay multiple ellipses on top of one another. In particular, a small black dot (a vanishing ellipse) indicates that the trivial solution $R_1 = R_2 = 0$ is stable.

The ellipse plots of Fig. 4 contain all the information in our results. As with the heat map in Fig. 2 and the mesh plots in Fig. 3, we see that when $a_2$ increases (from top to bottom rows), the upper left part of each graph shows a stronger $R_1$ response. In addition, from the ellipse plots in Fig. 4 it is clear that when the upstream crosstalk $b_1$ increases, the lower right part of each graph with a small feedback $f_2$ changes from having no response to having both $R_1$ and $R_2$ activated.

Figure 5 presents a different slice through the parameter space by showing $R_1$ and $R_2$ as functions of $a_2$ and $b_1$ in each panel, for selected values of $f_1$ and $f_2$ which vary between the panels. (The $f_1$ and $f_2$ values used for these plots are indicated by green squares in Fig. 4.) Thus, each panel allows us to move across the different panels in Fig. 4, showing how the crosstalk strengths change the responses at fixed feedback strengths. For a large $f_2$ and relatively small $f_1$ (Fig. 5 top left), the $R_1$ response can be activated by increasing $a_2$ even though $R_1$’s cognate feedback $f_1$ is weak. Similarly, in the case of high $f_1$ and low $f_2$ (Fig. 5 bottom right), increasing $b_1$ will activate not only the response $R_1$ as expected from a high $f_1$, but also the response $R_2$ despite weak $f_2$.

### Discontinuity and multistability at high cooperativity

**Figure 6:**
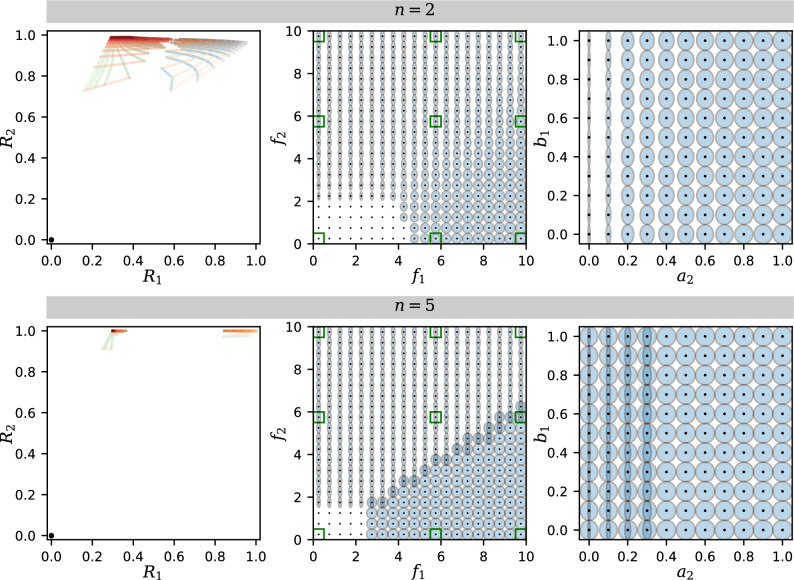
Examples of mesh and ellipse plots for higher cooperativity $n = 2$ and $n = 5$ (see Figs. S2 and S3 for detailed plots for $n=5$). Left column: $a_2 = b_1 = 0.3$, to be compared with the center panel of Fig. 3 under the same color scheme. Middle column: $a_2 = b_1 = 0.3$, to be compared with the center panel of Fig. 4. Right column: $f_1 = 9.8$ and $f_2 = 5.8$, to be compared with the mid-right panel of Fig. 5. All panels have $a_1 = b_2 = 1$.

Crosstalk can not only couple the two responses, as described above, but also restrict the joint responses to just a few distinct states. This can be observed for higher levels of cooperativity *n*. As examples, we show the results for $n = 2$ (Fig. 6 top row), which matches some experimental estimates^25^, and for $n = 5$ (Fig. 6 bottom row), which represents a high cooperativity limit. The mesh of Fig. 3 breaks up into multiple tight clusters (Fig. 6 left column). As a result, there are only three distinct stable states that exist: The on state is characterized by activation of both $R_1$ and $R_2$; the half-on state has $R_2$ activated and $R_1$ partially activated; the off state has no activation of either $R_1$ or $R_2$. Due to the asymmetric, hierarchical positioning of the pathways, there is no fourth state where $R_1$ is active while $R_2$ is inactive.

**Figure 7:**
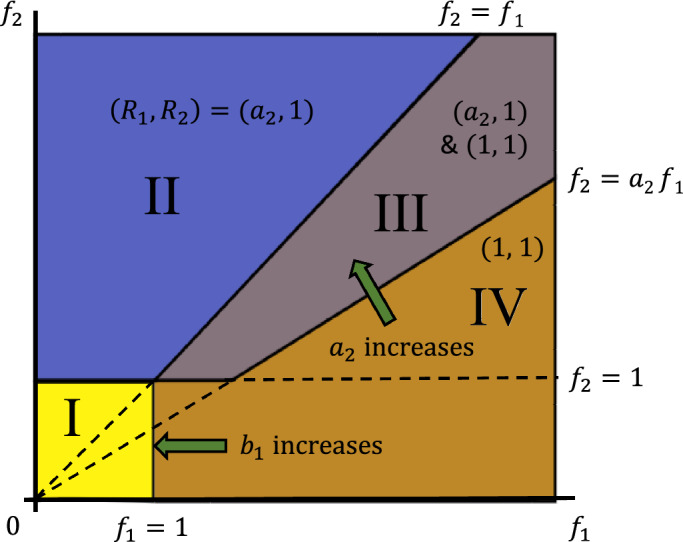
Phase diagram showing which states of $(R_1, R_2)$ are permitted for different combinations of $f_1$ and $f_2$ values (in addition to the trivial solution $R_1 = R_2 = 0$), in the limit of high cooperativity $n \rightarrow \infty$. (I) there is only the trivial solution - $R_1$ and $R_2$ both off; (II) $R_2$ is on, $R_1$ is partially on (proportional to $a_2$); (III) $R_2$ is on, $R_1$ can be either fully or partially on; (IV) both $R_1$ and $R_2$ are fully on.

As can be seen from Fig. 6 (middle column), at high cooperativity the responses $R_1$ and $R_2$ no longer change smoothly with the feedback strengths $f_1$ and $f_2$, but switch discontinuously along certain boundaries in the $(f_1, f_2)$ parameter space (see also Fig. S2 for $n=5$). In the limit $n \rightarrow \infty$, the phase diagram of Fig. 7 is obtained (see “Methods”), where each region of the parameter space permits different types of solutions to Eq. (2). In Region I, both feedbacks $f_1$ and $f_2$ are too small to activate a response, allowing only the trivial solution with $R_1, R_2$ both off. In Region II, with high $f_2$ and relatively low $f_1$, there exists an additional half-on state, with the upstream $R_2$ fully activated and the downstream $R_1$ only partially active. In Region IV, $f_1$ and $f_2$ are both sufficiently large to allow simultaneous activation of both responses $R_1$ and $R_2$, i.e., a fully on state instead of half-on. Between regions II and IV is region III, which allows both the half-on state and the fully on state, in addition to the off state.

Importantly, changes in the crosstalk strengths cause the boundaries in the phase diagram to move, because crosstalk allows the upstream and downstream feedback loops to activate each other. The sloped boundary between Region III and IV depends on the crosstalk parameter $a_2$: as $a_2$ increases, this boundary rotates counterclockwise around the origin, expanding Region IV and reducing Region III. In addition, the value of $R_1$ in Region II & III also increases with $a_2$. Similarly, the vertical boundary between Regions IV and I depends on $b_1$: if $b_1$ decreases to 0, this boundary moves all the way to the right, removing the small-$f_2$ portion of Region IV.

## Discussion

The model studied here does not contain all the ingredients of any quorum sensing pathway and is not intended as a completely faithful representation of *V. fischeri* LuxRI and AinRS pathways. It does however capture several common properties of quorum sensing networks: (1) multiple signals and cognate receptors drive multiple regulated outputs; (2) these pathways are functionally linked (sequentially in *V. fischeri*); (3) the pathways crosstalk through the interaction of signals with non-cognate receptors; (4) both pathways are regulated with positive feedback. The model offers insight into how the strength of crosstalk interacts with these architectural properties to alter the behavior of a quorum sensing system.

### The meaning of crosstalk

Quorum sensing pathways frequently employ multiple ligand-receptor pairs with limited binding specificity, where the “promiscuity” of this binding is a highly tunable or evolvable property^4,11,19^. Previous authors have investigated behavior of signaling pathways subject to this and other mechanisms of crosstalk; these include weak selectivity in ligand affinities at a cell surface receptor^16^, multiple ligand-receptor channels that merge to control a single regulated output^13,24^, or a receptor that has multiple sensing states and outputs that are associated with binding of different ligands^14^.

The ubiquity and tunability of crosstalk in quorum sensing systems raise the question of how even weak crosstalk may enhance the function of a multi-signal system. For example, a limited amount of crosstalk between two signaling pathways can in principle enhance the ability to measure the input signal concentrations^16^. It may also provide some benefit in suppressing early response^25^. Therefore, rather than consider a mechanistic model that embeds strong crosstalk into the topology, we study a model where distinct signaling pathways are coupled through tunable crosstalk parameters: crosstalk strength can range from a perturbation that weakly couples the two pathways to a strong link that drives two regulated outputs in tandem.

### Crosstalk through cross-binding or cross-activation

Our analysis highlights the importance of distinguishing between two different aspects of crosstalk that occurs when a receptor interacts with its non-cognate ligand (signal): One is cross-affinity, or the lack of specificity in binding, of a signal by the non-cognate receptor (characterized by parameter *b* in Eq. 1c). The other is the ability of the resulting non-cognate complex to cross-activate the regulated pathway (characterized by parameter *a*). If the receptor bound by the non-cognate signal is ineffective at promoting transcription, the result is competitive inhibition of the receptor. Mathematically, in Eq. (1c), the inhibition is due to $S_2$ appearing in the denominator of the expression for $R_1$. Thus, cross-binding allows the excitatory signal of one channel to inhibit the non-cognate response. On the other hand, if the receptor bound by a non-cognate signal can still promote transcription to some level, then there is cross-activation of the response, especially when the cognate signal is absent. Mathematically, when $S_1 = 0$, Eq. (1c) allows $R_1$ to be activated by signal $S_2$, although at a lower saturating level governed by $a_2$ (see “Methods”). Thus, the activation (*a*) and binding (*b*) components of crosstalk allow a response to be either activated or inhibited respectively by the non-cognate signal, when its own cognate signal is weak.

The dual effect of the non-cognate signal is observed in *V. fischeri*, where both C8-HSL and 3OC6-HSL are able to form an activating complex with LuxR. Because the C8-HSL-LuxR complex is less efficient at inducing *lux*, crosstalk from the *ainS/R* pathway inhibits the activating effect of 3OC6-HSL during the growth of a colony^26,27^. Although the C8-HSL autoinducer can stimulate luminescence at low concentrations of 3OC6-HSL^25^, at high 3OC6-HSL concentrations, the addition of C8-HSL reduces luminescence through the competition effect^28^.

### Weak crosstalk expands dynamic range

The extreme case of *V. harveyi*, where two signals merge to drive a single regulated output, is an example of two dimensions of signal input leading to a one-dimensional regulatory output. In *V. fischeri* the strength of crosstalk is evidently tunable between strains, as the HSL specificity of the LuxR receptor is strain-dependent. Further, both the *lux* regulatory region and the AinS/AinR system exhibit much greater sequence divergence between strain isolates of *V. fischeri* than is typical of the rest of the genome^29^. These findings suggest that the quorum sensing pathways in *V. fischeri* are under strong, strain-dependent selection pressures with consequences for *lux* control and associated crosstalk.

What benefits can different strains gain by tuning weak crosstalk interactions between the two pathways? Figure 3 shows graphically how crosstalk strength modulates the space of system outputs. A sensing system with two fully independent outputs such as $R_1$ and $R_2$ has in principle a two-dimensional space of outputs. In the limit of strong crosstalk these two independent outputs are collapsed to fall along the same, one-dimensional arc. Thus, fine tuning of the strength of crosstalk between the signal paths can define the dimensionality, shape, and extent of the response region in Fig. 3, tuning the dual output response to $f_1$ and $f_2$ along a continuum from orthogonality to tandem or ‘funneled’ control. Funneled control may be beneficial when there is a risk that quorum interference is removing one signal from the environment, so that “OR” sensing is desirable; orthogonality will be advantageous when multiple phenotypic behaviors need to be controlled by the dual-signal system. Crosstalk allows some compromise between these two limiting behaviors.

### Role of feedback

With both nonlinearity and feedback present in the model, one may expect to observe multistability, i.e., more than one steady state may be stable given the same parameter values. Especially in the limit of high cooperativity, we observe multistable states in several regions of the parameter space. Although multistability normally requires $n \ge 2$, in the presence of crosstalk there is multistability even for $n = 1$, such as when $b_1 > 0$ and $f_1$ is high but $f_2$ is low (Fig. 5 bottom right). This is because the coupling between the two feedback loops results in stronger nonlinearity than in a single feedback loop, allowing multistability (see “Methods”).

Experiments however find few clear examples of multistability in quorum sensing, which would be indicated by a multimodal distribution of corresponding phenotypes. With some exceptions (usually based on synthetic or ‘rewired’ pathways^30–32^), multistability is rarely observed in Gram negative quorum systems. Generally the highly diffusible HSL signals, together with positive feedback synthesis, act intercellularly to lock the entire population into an on-state. Because of extracellular accumulation of autoinducer, the off-state becomes increasingly unfavorable or unlikely compared to the on-state. Multistability in quorum sensing circuits is more evident when the signal feedback at the individual cell level is strengthened, either by circuit redesign^31^ or because the signal is able to act intracellularly, allowing isolated cells to autoactivate^33^.

Another factor that makes it difficult to observe multistability in individual cells is that experiments often fix the extracellular signal concentrations at defined levels, or use strains in which feedback has been broken by deletion of the signal synthases. Thus, the difference between the multistability in experimental conditions and in our model highlights the important distinction between keeping the signals constant and letting them “float” according to the feedback. To observe multistability one must allow the signal levels to float either upward or downward, as natural systems do. However, in many laboratory experiments, the signal levels are externally controlled by supplying those molecules at known concentrations. As a result, multistability is suppressed and noise in gene expression is a much more significant source of heterogeneity^34,35^.

### New motifs: “jump-start” and “push-start”

**Figure 8:**
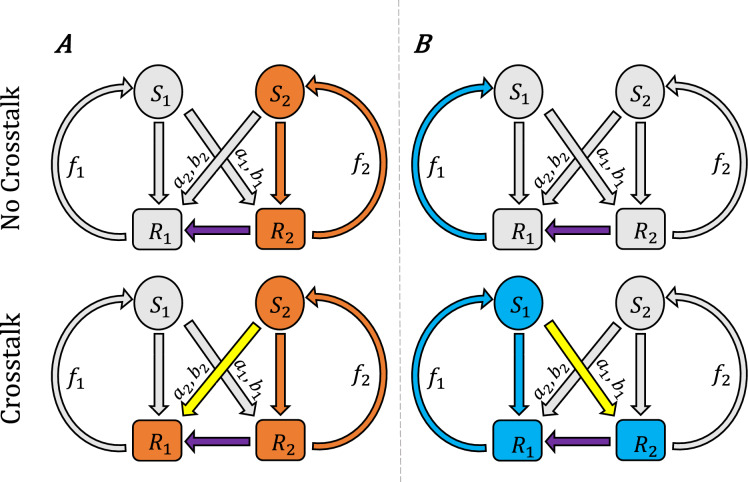
Crosstalk provides two new mechanisms for activating both pathways without requiring strong feedback in both. In the “jump start” scenario (left), even when the feedback $f_1$ is weak but $f_2$ is strong, the downstream-directed crosstalk ($a_2, b_2$) allows the $S_2$-$R_2$ pathway to activate and drive the downstream $R_1$ response. In the “push start” scenario (right), when the feedback $f_2$ is weak but $f_1$ is strong, the upstream-directed crosstalk ($a_1, b_1$) allows the $R_2$ response to be driven by the $S_1$-$R_1$ pathway, activating both $R_1$ and $R_2$.

Our results show that crosstalk can allow either of the feedback loops – upstream or downstream – to activate the other loop, via separate mechanisms driven by $a_2$ and $b_1$ respectively. As illustrated schematically in Fig. 8A, the first mechanism is engaged when the feedback strength $f_1$ is low but $f_2$ is high (Region II in Fig. 7). A large $f_2$ turns on the $S_2$-$R_2$ feedback loop, but without crosstalk the $R_1$ response is off due to a small $f_1$. Strengthening the downstream-directed crosstalk $a_2$ allows the upstream $S_2$-$R_2$ feedback loop to also activate the downstream $R_1$ response (Fig. 8A). This is roughly analogous to the “jump-start” of a combustion engine, where an upstream system consisting of a battery, alternator and starter motor is mechanically coupled to a downstream system consisting of the combustion engine and flywheel: activating the upstream branch by energizing the starter system turns the motor which then starts the combustion engine.

The second mechanism, shown in Fig. 8B, applies when $f_1$ is large but $f_2$ is small (lower part of Region IV in Fig. 7). In the absence of crosstalk, even a large $f_1$ cannot turn on $R_1$, because it depends on the upstream response $R_2$ that is off due to a small $f_2$. However, sufficient upstream-directed crosstalk $b_1$ can allow the downstream $R_1$-$S_1$ feedback to drive the upstream $R_2$ and activate both $R_1$ and $R_2$ (Fig. 8B). This has a rough analogy in the “push-start” of a combustion engine with a dead starter battery: the mechanical coupling from the engine crankshaft to the upstream battery/alternator system allows an energy input at the wheels to turn the engine, which then turns the alternator, replacing the role of the battery and activating the downstream (engine) and upstream (alternator/battery) systems.

**Table 1:**
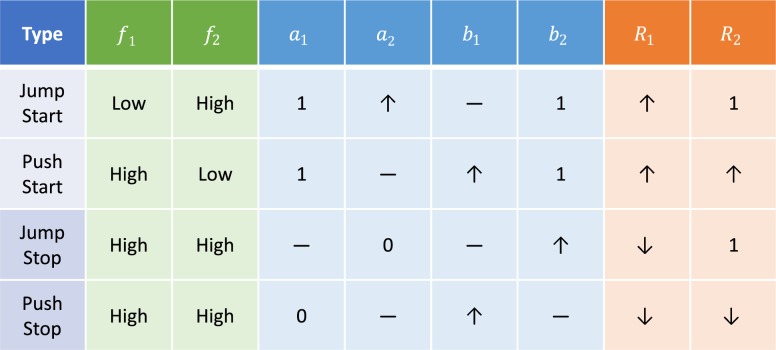
Modes of activation and deactivation through crosstalk between two coupled quorum sensing pathways.

The “jump start” and “push start” modes activate the system as illustrated in Fig. 8, whereas the “jump stop” and “push stop” modes shut down the system. The $f_1, f_2$ columns show the feedback conditions required for each mode, while the $a_1$-$b_2$ columns show how the crosstalk parameters must be configured, to generate the outputs shown in the $R_1, R_2$ columns. A dash means the parameter does not strongly impact the behavior.

**Figure 9:**
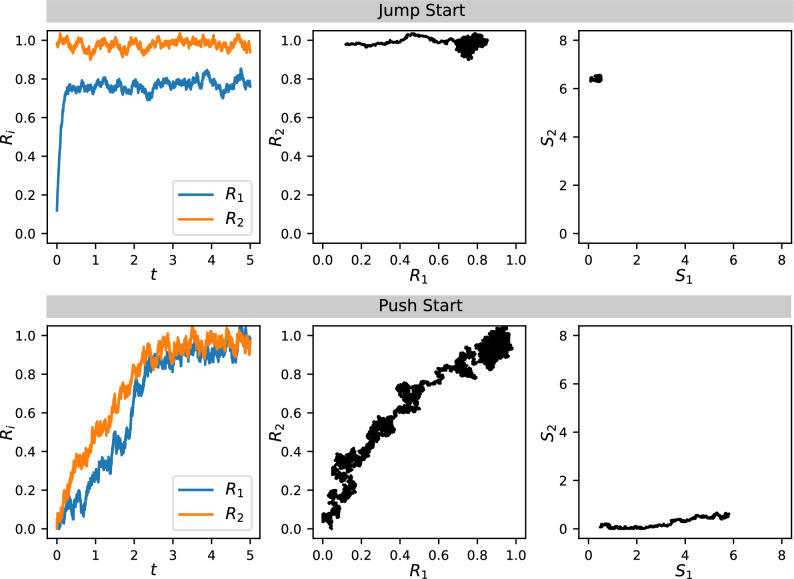
Simulation of the jump-start and push-start processes using a dynamical system with noise (see “Methods”). Top row: Jump-starting from an initial state with $R_1$ off by having a large crosstalk $a_2$. Parameters are $f_1 = 0.6$, $f_2 = 6.6$, $a_1 = 1$, $a_2 = 0.8$ (initially 0.1), $b_1 = 0.5$, $b_2 = 1$, $n = 2$. Bottom row: Push-starting from an initial state with $R_1$ and $R_2$ both off by having a large crosstalk $b_1$. Parameters are $f_1 = 6.6$, $f_2 = 0.6$, $a_1 = 1$, $a_2 = 0.5$, $b_1 = 0.8$ (initially 0.1), $b_2 = 1$, $n = 2$.

To illustrate these mechanisms, we simulate a dynamical system corresponding to our model (Eqs. (12–15) in “Methods”). The initial values are chosen to be the steady states for the system with small crosstalk, i.e., low $a_2$ for jump-start, and low $b_1$ for push-start. Then we switch on large values for these crosstalk parameters and run the dynamical system until it reaches a new steady state. Our simulation also includes random noise in the dynamics to show the stability of the steady states. For jump-start (Fig. 9 top row), the initial state is such that $R_2$ is already on but $R_1$ is off, due to a low $f_1$ and a small $a_2$. We then reset $a_2$ to a large value and the dynamics take the system to a new steady state where $R_1$ turns on. Note that $S_1$ remains low because $R_1$ is turned on by the crosstalk $a_2$, not by the feedback $f_1$. For push-start (Fig. 9 bottom row), both $R_1$ and $R_2$ are off in the initial state with a low $f_2$ and a small $b_1$. When $b_1$ is switched to a large value, the noisy dynamics allow the system to escape the off state and turn on both $R_1$ and $R_2$. In this case $S_2$ remains low because the responses are turned on by the crosstalk $b_1$ instead of the feedback $f_2$.

These two mechanisms can be thought of as new regulatory motifs that could be embedded within larger gene regulatory networks. The essence is that two feedback loops, linked by crosstalk, are positioned upstream and downstream from each other. Crosstalk between the feedback loops allows activation through the jump-start (upstream feedback loop activating downstream response) and push-start (downstream feedback loop activating both responses) behaviors. In addition to these motifs (where we set $a_1 = b_2 = 1$), there are other interesting behaviors when all the crosstalk parameters are considered, as summarized in Table 1. In particular, we have the opposite of the jump-start and push-start, which could be called “jump-stop” and “push-stop”, where crosstalk from one branch can inhibit the function of the other branch.

## Conclusion

Although crosstalk in engineering contexts refers to an undesired leakage of information between separate communication channels, in the context of biological sensing it can provide additional mechanisms for the control or activation of coupled feedback systems that are ubiquitous in quorum sensing pathways. In our analysis crosstalk appears to provide a route for switching individual feedback circuits on or off without relying entirely on extracellular signal concentrations as in typical interpretations of quorum sensing. Our findings are based on analyzing the equilibrium states of the feedback circuits that are coupled through crosstalk. How crosstalk affects the kinetics of the system as it approaches the equilibrium is likely an important component of its biological role, which remains to be studied.

The variability of crosstalk strength across different quorum sensing systems and even across strains of the same bacterial species suggests that the new mechanisms for activating the feedback circuits can be exploited through evolutionary tuning of the crosstalk strength. For example, crosstalk could provide a form of redundancy so that a pathway can still be activated when the signal is being inhibited, such as by quorum interference: If a sabotaging species removes the signal $S_1$ from the environment, or creates an interfering signal $S_3$ that saturates the $S_1$ receptor, the jump-start mechanism may ensure that the downstream response $R_1$ can still be activated. Likewise, the push-start mechanism may protect against external interference with the signal $S_2$ of the upstream response $R_2$.

It may also be possible that crosstalk strengths could be tuned on short timescales by cellular processes. For example, the cross-binding strength *b* could be affected by allosteric interactions with modifier proteins, while the cross-activation efficiency *a* could be controlled by other ligands or post-translational regulation. If crosstalk was variable in real time, instead of over evolutionary timescales, then it could be a very significant mechanism for control. It would be interesting if experiments could show that crosstalk strengths are variable within the same species and under different environmental conditions, allowing jump-start and push-start in real time. This could even lead to community-level phenomena, such as one species triggering the quorum sensing pathway of another by tuning their crosstalk strengths, i.e., an “interspecies jump-start”.

## Methods

### Formulating the mathematical model

We consider a quorum sensing system in which two autoinducer signals drive the activity of two largely distinct, but coupled, regulatory pathways. In a simplified picture, where the two pathways operate without crosstalk, the autoinducer binds to a cognate receptor and the bound complex promotes the expression of a corresponding set of genes, including one that encodes the autoinducer itself. The specific mechanisms of signal transduction are variable, and may involve an intracellular receptor that binds the signal to form a transcriptional activator, or a membrane bound receptor that controls a phosphorylation cascade.

We think of the signals $S_i$ as the concentrations of the two autoinducer species, and the responses $R_i$ as the expression levels of the corresponding quorum regulated genes. We model the gene expression level as a function of the autoinducer concentration using a binding-equilibrium expression that resembles the Michaelis–Menten and Hill equations,

3 $$R_i = g_i \, \frac{k_i S_i^n}{1 + k_i S_i^n} ,$$

where $g_i$ is the maximum level at saturation and $k_i$ represents the binding affinity of the autoinducer. For each pathway the expression level $R_i$ of quorum sensing genes determines the production of the corresponding autoinducer $S_i$, through positive regulatory feedback. For simplicity, we assume that:

4 $$S_i= f_i R_i$$

where $f_i$ is the strength of feedback and depends on the diffusion and degradation of the autoinducer molecules.

Crosstalk between the two quorum sensing pathways occurs when the autoinducer of one pathway can modulate gene expression in the other pathway. For example, a non-cognate autoinducer may bind to the receptor with some affinity, producing a resultant complex that promotes gene expression with some efficiency. Thus, the gene expression level will depend on the concentration of both autoinducers. We model such dependence by modifying Eq. (3) to:

5 $$R_i= g_i \, \frac{k_i S_i^n + a_j b_j S_j^n}{1 + k_i S_i^n + b_j S_j^n}$$

Here *j* is the label for the non-cognate signal – the $b_j S_j^n$ term in the denominator represents competitive binding by the non-cognate autoinducer, and the $a_j b_j S_j^n$ term in the numerator represents cross-activation by the non-cognate complex. The parameter $b_j$ represents the binding affinity of the non-cognate autoinducer to the receptor, and $a_j$ represents the promotion efficiency of the non-cognate complex. This form of dependence on multiple signals is fairly general as it can be derived for different, common quorum sensing system architectures^24,25^. Detailed derivation of these equations for the specific pathways in *V. fischeri* is described in the Supplementary Material.

We now incorporate some more details of the circuit that is present in the *Vibrio fischeri* example. In that system, pathway 2 is upstream of pathway 1 (Fig. 1A), so that the expression level of $R_1$ depends on that of $R_2$ (Fig. 1B). This is modeled by simply making $R_1$ proportional to $R_2$,

6 $$R_1= g_1 \, \frac{k_1 S_1^n + a_2 b_2 S_2^n}{1 + k_1 S_1^n + b_2 S_2^n} \, R_2$$

7 $$R_2= g_2 \, \frac{a_1 b_1 S_1^n + k_2 S_2^n}{1 + b_1 S_1^n + k_2 S_2^n}$$

We may remove the parameters $g_i$ and $k_i$ by rescaling $S_1^n \leftarrow S_1^n / k_1$, $S_2^n \leftarrow S_2^n / k_2$, $R_1 \leftarrow g_1 g_2 R_1$, and $R_2 \leftarrow g_2 R_2$, and redefining parameters $b_1 \leftarrow b_1 k_1$, $b_2 \leftarrow b_2 k_2$. After such rescaling the equations finally become:

8 $$S_1= f_1 R_1$$

9 $$S_2= f_2 R_2$$

10 $$R_1= \frac{S_1^n + a_2 b_2 S_2^n}{1 + S_1^n + b_2 S_2^n} \, R_2$$

11 $$R_2= \frac{a_1 b_1 S_1^n + S_2^n}{1 + b_1 S_1^n + S_2^n}$$

### Numerical methods for finding solutions

To find the solutions to Eqs. (8–11), we consider a system of differential equations whose equilibrium states are the solutions to those equations above. The differential equations we use are:

12 $$\frac{dS_1}{dt}= \frac{1}{\tau _S} \big ( f_1 R_1 - S_1 \big )$$

13 $$\frac{dS_2}{dt}= \frac{1}{\tau _S} \big ( f_2 R_2 - S_2 \big )$$

14 $$\frac{dR_1}{dt}= \frac{1}{\tau _R} \bigg ( \frac{S_1^n + a_2 b_2 S_2^n}{1 + S_1^n + b_2 S_2^n} \, R_2 - R_1 \bigg )$$

15 $$\frac{dR_2}{dt}= \frac{1}{\tau _R} \bigg ( \frac{a_1 b_1 S_1^n + S_2^n}{1 + b_1 S_1^n + S_2^n} - R_2 \bigg )$$

where the timescales are set to $\tau _S = \tau _R = 1$ for simplicity. For each set of parameter values, we integrate these equations using the SciPy function solve_IVP() for 500 time units, starting from random initial values. This should bring the variables sufficiently close to a local equilibrium. We then refine the result using SciPy’s root-finding function scipy.optimize.root(), with the previous result as the initial guess. Alongside this, we calculate the Jacobian matrix of our system of equations to verify that the equilibrium that we have found is stable. In order to find all potential solutions for a given set of parameters, we repeat this process 100 times with different random initial values and eliminate any duplicate solutions. To generate Figs. 2, 3, 4, 5 and 6 in the main text, we scan over the parameter space and apply the above procedure at every grid point in the parameter space. For the stochastic simulations in Fig. 9, we use $\tau _S = 1$ and $\tau _R = 0.1$; a random Gaussian noise is added to each equation with an amplitude $\sigma = 20$. The stochastic differential equations are integrated using the Euler–Maruyama method with a time step of $\Delta t = 0.0001$.

### Analytic results at $n \rightarrow \infty$

In the limit $n \rightarrow \infty$, the solutions to Eq. (2) can be found using the following arguments:

- If both $R_1$ and $R_2$ are small, such that $f_1 R_1 < 1$ and $f_2 R_2 < 1$, then the right-hand side (RHS) would lead to $R_1, R_2 \rightarrow 0$. Indeed, $R_1 = R_2 = 0$ is always a solution.
- If $f_2 R_2 > 1$ and $f_2 R_2 > f_1 R_1$, then the RHS gives $R_2 = 1$ and $R_1 = a_2$. To be consistent, we need $f_2 > 1$ and $f_2 > a_2 f_1$, which corresponds to Regions II and III in Fig. 7.
- If $f_1 R_1> f_2 R_2 > 1$, then the RHS gives $R_1 = R_2 = 1$. To be consistent, we need $f_1> f_2 > 1$, which corresponds to Region III and part of Region IV in Fig. 7.
- If $f_1 R_1> 1 > f_2 R_2$, then the result depends on $b_1$. For $b_1 > 0$, the RHS gives $R_1 = R_2 = 1$ as in (c), which corresponds to the $f_1> 1 > f_2$ part of Region IV in Fig. 7. But for $b_1 = 0$, the RHS gives $R_1 = R_2 = 0$, which means this part is merged into Region I.

Taken together, the above arguments imply that:

(i) In Region I, the only solution is $(R_1, R_2) = (0, 0)$.
(ii) In Region II, both solutions (0, 0) and $(a_2, 1)$ are possible.
(iii) In Region III, three solutions are possible, (0, 0), $(a_2, 1)$, and (1, 1).
(iv) In Region IV, two solutions are possible, (0, 0) and (1, 1).

These different regions are shown in Fig. 7.

In some regions of interest, approximate solutions can be obtained to understand the behavior of the system. In the case where $f_1$ is small (left part of Region II), we can approximate that $S_1 \approx 0$. This allows us to ignore the effect of $S_1$ and simplify the equations to:

16 $$S_2= f_2 R_2$$

17 $$R_2= \frac{S_2}{1+S_2}$$

18 $$R_1= \frac{a_2 b_2 S_2}{1 + b_2 S_2} R_2$$

In this region, the $R_1$ response is completely controlled by the crosstalk coming from the $S_2$-$R_2$ feedback loop, which is the “jump-start” scenario. The activation level of $R_1$ is controlled by the parameter $a_2$, while its dependence on $b_2$ is weak as long as the feedback $f_2$ is strong enough to highly express $S_2$ (i.e., $f_2 > 1/b_2$).

Similarly, when $f_2$ is small (lower part of Region IV), we can ignore $S_2$ and simplify the equations to:

19 $$S_1= f_1 R_1$$

20 $$R_1= \frac{S_1}{1 + S_1} R_2$$

21 $$R_2= \frac{a_1 b_1 S_1}{1+b_1 S_1}$$

In this case, when $a_1$ and $b_1$ are not too small, there is a feedback loop $S_1$-$R_2$-$R_1$ that can activate both responses $R_1$ and $R_2$ to a level controlled by $a_1$, which is the “push-start” scenario. For $a_1 = 1$, both responses can be fully activated as long as the feedback $f_1$ is strong enough ($f_1 > 1 / b_1$). In other words, the responses can be turned on by tuning the parameter $b_1$ above a threshold $\sim 1 / f_1$. Note that if we eliminate $R_2$ from Eqs. (20, 21), then $R_1$ as a function of $S_1$ is more nonlinear than a Hill’s function with $n=1$.

### Supplementary Information

Supplementary Figures.

## Data Availability

All data generated or analysed during this study are included in this published article.
