# Supplementary material for: Crosstalk enables mutual activation of coupled quorum sensing pathways through “jump-start” and “push-start” mechanisms
Source: Sci Rep. 2023 Nov 6;13:19230. doi: 10.1038/s41598-023-46399-z (PMC10628186; doi:10.1038/s41598-023-46399-z)
Supplement: Supplementary file 1 — Supplementary Figures. [file 41598_2023_46399_MOESM1_ESM.pdf]

Crosstalk enables mutual activation of coupled quorum sensing pathways through "jump-start" and "push-start" mechanisms

Joseph George Sanders[1], Hoda Akl[1], Stephen J Hagen[1], and BingKan Xue[1,*]

[1]Department of Physics, University of Florida, Gainesville, FL 32611, USA
[*]b.xue@ufl.edu

Supplementary Text

Derivation of crosstalk equations based on the specific *V. fischeri* circuit

The signaling system with two coupled pathways modeled in the main text is based loosely on the *luxIR* and *ainRS* quorum sensing pathways in *V. fischeri*. Here we go into some biochemical detail to motivate equation (5), which describes the dependence of the response variables R_i on both signals. In the case of *V. fischeri*, the two signals S_1 and S_2 may represent the autoinducers 3OC6-HSL and C8-HSL respectively, and the two responses R_1 and R_2 may be analogous to the expression of the *luxI* and *ainS* genes that encode the synthases of the autoinducers respectively.

For pathway 1, the 3OC6-HSL binds reversibly to the transcription factor *LuxR* with an association constant k_1. In addition, *LuxR* has a cross-interaction with C8-HSL, with an association constant b_2. Let $[LuxR_0]$ and $[LuxR]$ be the concentration of total and unbound *LuxR*, then:

$$\begin{aligned}[LuxR_0] &= [LuxR] + [\text{3OC6-}LuxR] + [\text{C8-}LuxR] \\ &= [LuxR] + k_1[\text{3OC6HSL}][LuxR] + b_2[\text{C8HSL}][LuxR]\end{aligned}$$

Both complexes 3OC6-*LuxR* and C8-*LuxR* can activate the transcription of the *luxI* gene, with different efficiencies a_1 and a_2. If the promoter is not saturated, the transcription rate will be linear in both complexes:

$$a_1[\text{3OC6-}LuxR] + a_2[\text{C8-}LuxR] = \frac{a_1k_1[\text{3OC6HSL}] + a_2b_2[\text{C8HSL}]}{1 + k_1[\text{3OC6HSL}] + b_2[\text{C8HSL}]}[LuxR_0] \tag{S1}$$

The total amount of *LuxR* depends on the expression of *LitR*, which is a transcription factor for the *luxR* gene, as well as for the *ainS* gene in the other (upstream) quorum sensing pathway. Therefore, we assume that $[LuxR_0] \propto R_2$. Writing [3OC6] as S_1 and [C8] as S_2, we have:

$$\frac{dR_1}{dt} = \frac{a_1k_1S_1 + a_2b_2S_2}{1 + k_1S_1 + b_2S_2}\lambda R_2 - \mu R_1 \tag{S2}$$

where μ is the loss or degradation rate. At equilibrium, we find:

$$R_1 = g_1\frac{k_1S_1 + a_2b_2S_2}{1 + k_1S_1 + b_2S_2}R_2 \tag{S3}$$

where we defined $g_1 \equiv a_1\lambda/\mu$ and redefined $a_2 \leftarrow a_2/a_1$.

If we assume a certain degree of multi-merization in the binding of HSL (either 3OC6 or C8) to *LuxR*, i.e.,

$$n\,\text{HSL} + n\,LuxR \leftrightarrow (\text{HSL-}LuxR)_n \tag{S4}$$

then the above equation will become:

$$R_1 = g_1\frac{k_1S_1^n + a_2b_2S_2^n}{1 + k_1S_1^n + b_2S_2^n}R_2 \tag{S5}$$

Experimental estimates of the coefficient n range roughly between 1–2 [25].

For pathway 2, the C8-HSL binds to a transmembrane receptor *AinR*, which is a histidine kinase that triggers a signal transduction through a phosphorelay protein *LuxU*. Binding of the HSL to the receptor eventually leads to the increase of the *LitR* level, which promotes the transcription of the *ainS* gene. It has been shown that the 3OC6-HSL can also promote the expression of *LitR*, although the mechanism has not been fully determined. In a homologous quorum sensing pathway in *V. harveyi*, *LuxU* can be phosphorylated by multiple receptors; the dependence of the shared output variable on the input from each autoinducer was modeled using a similar form to our Eq. (5) (see Eq. (4) in [24]).

Supplementary Figures

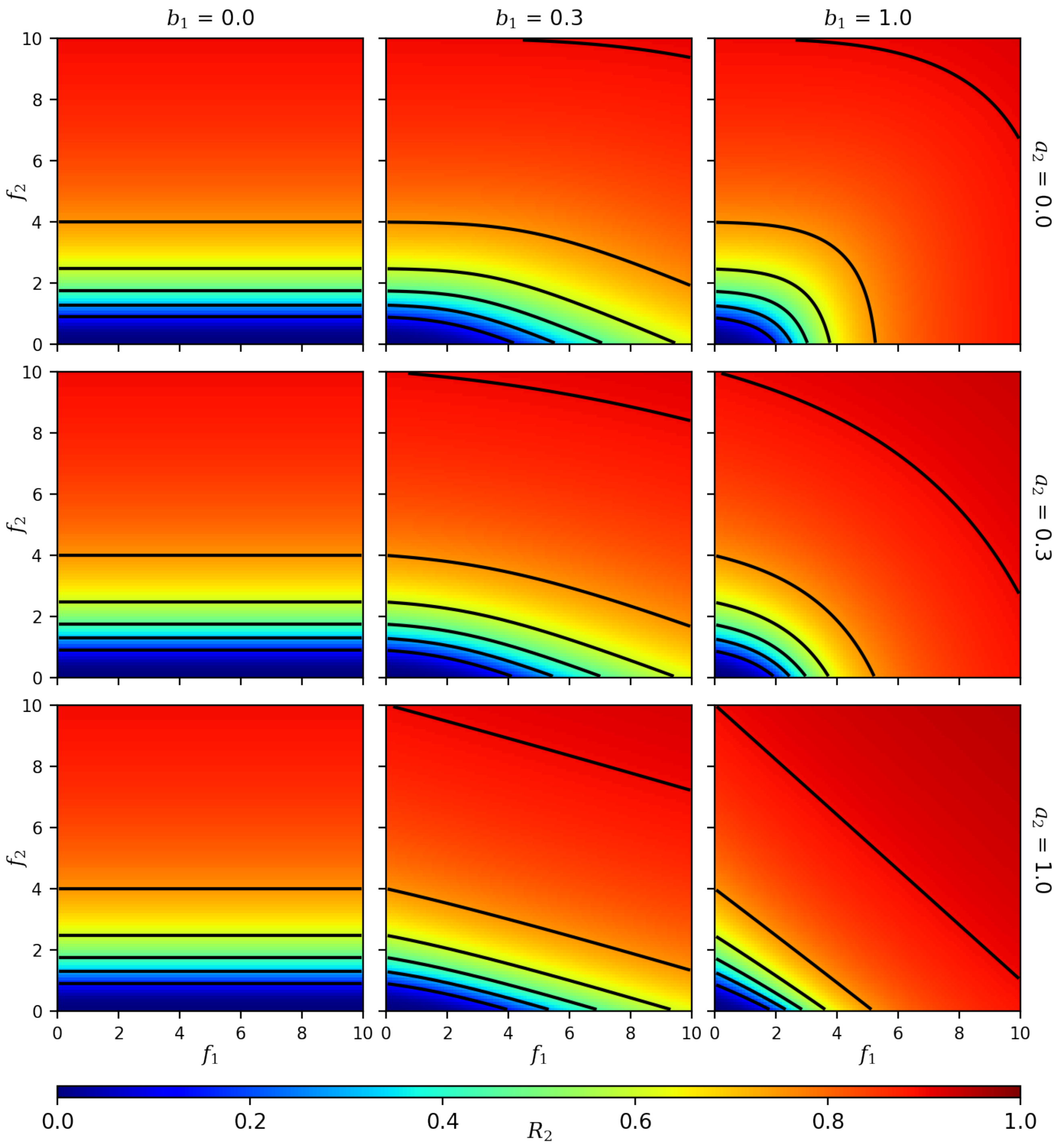

Figure S1. Heat maps showing the value of R_2 as a function of the feedback strengths f_1 and f_2 (compare with Figure 2 for R_1). Rows correspond to different values of the downstream-directed crosstalk activation strength a_2, whereas columns correspond to values of the upstream-directed crosstalk binding strength b_1 (all panels have $a_1 = b_2 = 1$, $n = 1$). Black lines are contours of constant R_2.

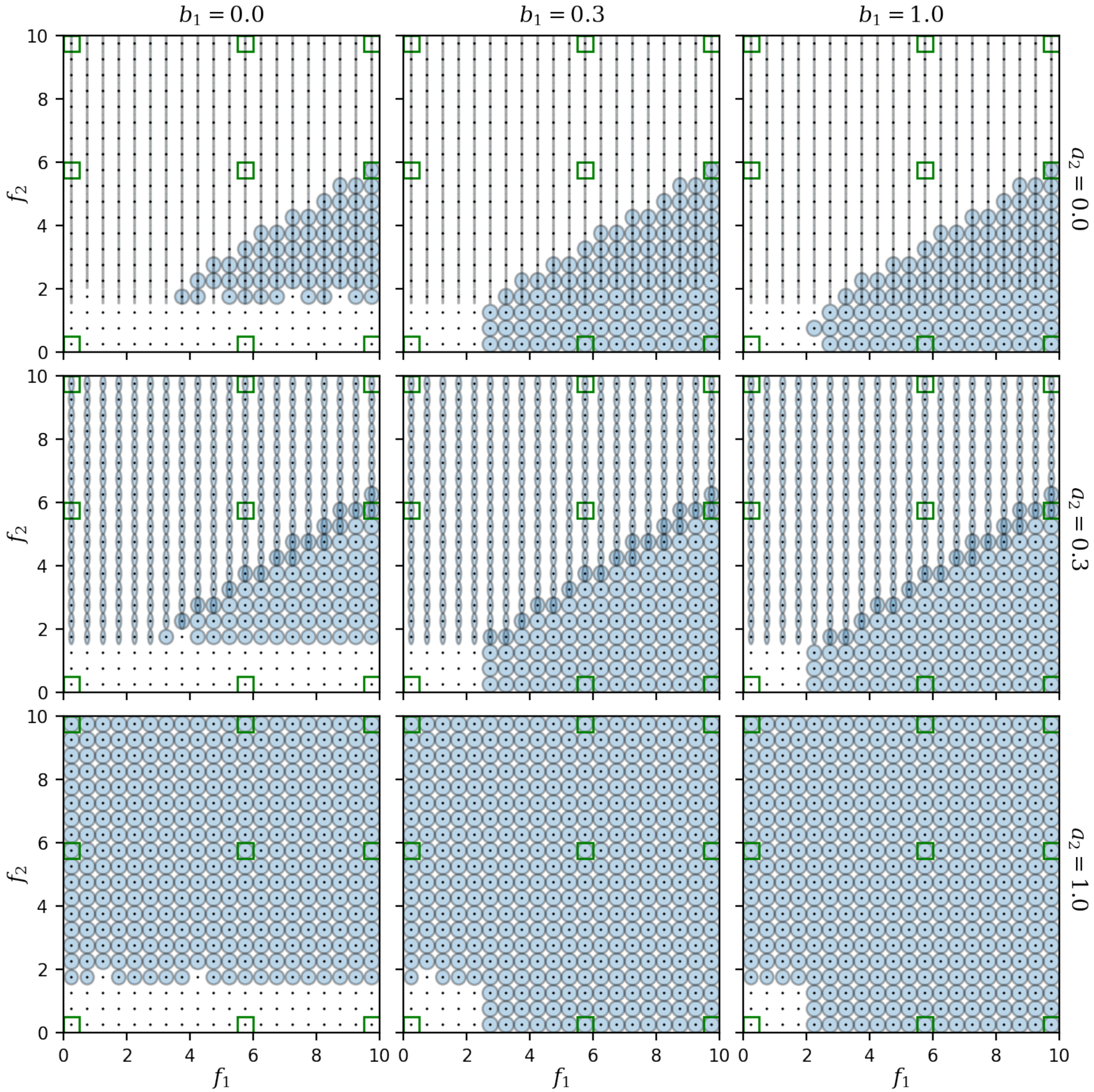

Figure S2. "Ellipse plots" showing the responses R_1 and R_2 as functions of the feedback strengths f_1 and f_2 for high cooperativity $n = 5$ (compare with Figure 4 for $n = 1$). The width and height of each ellipse represent the values of R_1 and R_2, respectively, at a given point in the (f_1, f_2) plane. Multiple ellipses overlaid on top of one another represent multiple stable solutions; a black point represents a vanishing ellipse with $R_1 = R_2 = 0$. Rows and columns represent different values of the crosstalk parameters a_2 and b_1, respectively (with $a_1 = b_2 = 1$). The f_1 and f_2 values marked in green are shown in Figure S3.

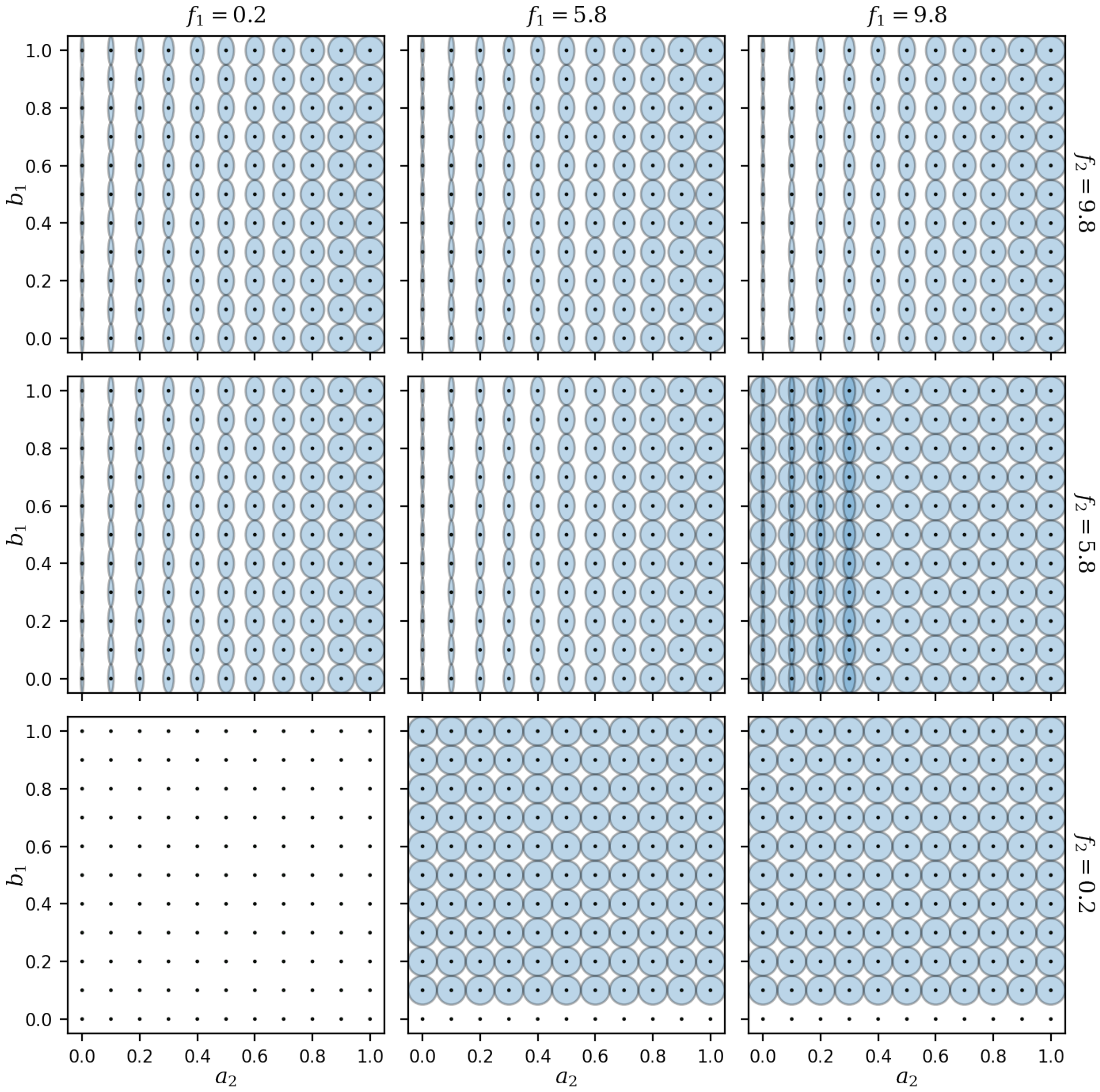

Figure S3. Dependence of the responses R_1 and R_2 on the crosstalk parameters a_2 and b_1 for high cooperativity $n = 5$ (compare with Figure 5 for $n = 1$). With the same parameters as in Figure S2, the width and height of each ellipse represent the values of R_1 and R_2, respectively. Rows and columns here represent different combinations of the feedback f_1 and f_2 (marked green in Figure S2). Multiple ellipses overlaid on top of one another represent multiple stable solutions; a black point represents a vanishing ellipse with $R_1 = R_2 = 0$.
